# Carbon Dioxide Elimination After Sodium Bicarbonate Administration as a Novel Method to Assess Cardiac Output: A Pilot Study

**DOI:** 10.7759/cureus.18621

**Published:** 2021-10-09

**Authors:** Ilan Keidan, Samantha Arzillo, Terrie Vasilopoulos, Erez Ben-Menachem, Nikolaus Gravenstein, Edward McGough

**Affiliations:** 1 Anesthesiology, University of Florida College of Medicine, Gainesville, USA; 2 Anesthesiology/Orthopedics and Rehabilitation, University of Florida College of Medicine, Gainesville, USA; 3 Anaesthesia, St. Vincent's Hospital, Sydney, AUS

**Keywords:** carbon dioxide, cardiac output, cardiopulmonary bypass, pulmonary circulation, sodium bicarbonate

## Abstract

Introduction

Cardiac output/pulmonary blood flow measurement is an important way to assess patients during the perioperative period, as well as patients who are critically ill. Current methods of assessing cardiac output have limitations. One indicator of cardiac output may be the expired carbon dioxide (CO_2_) partial pressure response to intravenous sodium bicarbonate (IVSB), which is rapidly converted to CO_2_.

Methods

We conducted an initial evaluation of the relationship between expired CO_2_ partial pressure and blood flow after a bolus of IVSB. To assess this relationship, we used a cardiopulmonary bypass circuit with predetermined blood flows in a laboratory trial and then assessed 18 patients undergoing surgery requiring cardiopulmonary bypass.

Results

For the laboratory portion of this pilot study, higher peak expired CO_2_, faster time to reach peak, higher area under the curve, and greater kurtosis of peak were observed at higher cardiac output flow rates, and higher mean expired CO_2_ was significantly associated with higher flow rates (p < 0.001). In the human study, higher mean (p = 0.023) and peak expired CO_2_ (p = 0.028) were both significantly associated with higher cardiac output flow rates.

Conclusions

This technique may be a way to intermittently assess cardiac output or improve accuracy when used in conjunction with other continuous output monitors.

## Introduction

Hemodynamic management of the perioperative or critically ill patient is fundamental to maintaining adequate tissue perfusion and organ homeostasis during physiological perturbations. Cardiac output measurement is key to understanding an individual’s oxygen delivery to end organs [1], and such advanced monitoring combined with “goal-directed” management strategies may improve outcomes [2]. Current methods of cardiac output measurement have limitations, such as invasiveness (e.g., a pulmonary artery catheter), reliance on sinus rhythm (e.g., arterial waveform analysis), or user dependence (e.g., esophageal Doppler) [3]. Furthermore, increasingly popular noninvasive methods such as pulse contour analysis of the arterial waveform have variable accuracy depending on the algorithm used [4].

The gold standard for cardiac output monitoring remains the pulmonary artery catheter and transpulmonary thermodilution [5]. Thermodilution is based on the principle of delivering an indicator (cold or heat injectate) and measuring its appearance at a distal location using a circulatory bed that receives the entire cardiac output (i.e., right heart thermodilution). Alveolar carbon dioxide (CO_2_) partial pressure is dependent on tissue CO_2_ production and alveolar ventilation; however, temporal changes are also dependent on changes in pulmonary blood flow (cardiac output). End-tidal CO_2_ (etCO_2_) has been shown to correlate with cardiac output [6]. Intravenous sodium bicarbonate (IVSB) is rapidly converted to CO_2_; therefore, it may act as a surrogate indicator of cardiac output. Significant increases in expired CO_2_ have been observed in response to an intravenous bolus of sodium bicarbonate [7].

During steady state, alveolar CO_2_ as well as etCO_2_ is determined by CO_2_ production and alveolar ventilation. CO_2_ that is produced in the periphery is delivered via the venous blood into the pulmonary circulation. Additional CO_2_ as is produced by the conversion of sodium bicarbonate to CO_2_ is also delivered by the same venous system and pulmonary flow. One factor that determines the rate at which CO_2_ appears in expired gas after IVSB is pulmonary blood flow.

We conducted an initial evaluation of the relationship between expired CO_2_ partial pressure changes after a bolus of IVSB and pulmonary blood flow in a biological model where the cardiac output is known. This study was undertaken in two parts: the first part used a cardiopulmonary bypass (CPB) circuit allowing a precise predetermined cardiac output and the second part was conducted in patients undergoing surgery requiring CPB.

## Materials and methods

Laboratory study

An adult CPB pump was primed with 700 mL of packed red blood cells and 1,000 mL of normal saline. The sweep flow of the gas flowing into the CPB oxygenator was fixed at 1 L/min to approximate a fixed minute ventilation. The flow of the CPB pump was varied from 1 to 6 L/min in 1 L/min increments. CO_2_ released from the oxygenator was sampled continuously using a Capnostream™ 20p Monitor with Microstream™ technology (Medtronic, Minneapolis, MN). Data were recorded every 20 ms via digital output, recorded on a laptop computer, and stored for offline analysis.

At each pump flow rate, 50 mL of 8.4% IVSB was rapidly injected as a bolus into the venous reservoir. After each injection, 3 minutes was allowed for the CO_2_ to equilibrate and be cleared from the system. Mean expired CO_2_, peak expired CO_2_ (mmHg), time to peak from injection (seconds), area under the curve (AUC), and kurtosis were analyzed. Mean expired CO_2_ was the average across all the 20-ms measurements (n = 1,776 for each flow rate). Thus, variability could be estimated for mean expired CO_2_; other measurements were reported as single values because only one run for each flow rate was performed. AUC was calculated using the trapezoidal method and quantified total etCO_2_ over time (MedCalc Software Ltd, Ostend, Belgium; https://www.medcalc.org; 2020). Kurtosis is an indicator of waveform distribution and quantifies sharpness of peak. CPB pumps were tested six times in incremental flows between 1 and 6 L/min; for each, an increase or decrease in flow was chosen randomly. Between each test, a period of continuous flow of 2.5 L/min was allowed for 5 minutes.

Clinical study

After University of Florida Institutional Review Board approval (IRB #201601877), 18 adult patients undergoing surgery requiring CPB were recruited and written informed consent from all participants was obtained. Patients were excluded if they were under 18 years of age, did not speak English as a primary language, or were enrolled in another study involving drug administration. Patients received general anesthesia, with drug selection and dosages at the discretion of the treating anesthesiologist. The patients were endotracheally intubated and cannulated for CPB (4,000 mL CAPIOX® Advance Reservoir, TERUMO, Tokyo, Japan).

Once the patient was stable on CPB and on a constant pump flow with a stable sweep gas flow (2 L/min), a baseline arterial blood gas was obtained. CO_2_ was continuously measured using the same CO_2_ monitor and computer as in the CPB bench study. The CO_2_ monitor was attached to the gas outlet port of the oxygenator. The measured flow on the CPB pump was held constant and recorded. Subsequently, 50 mL of 8.4% IVSB was rapidly injected into the venous reservoir. Data collection continued and were recorded for 90 seconds.

Measured variables included change in mean expired CO_2_, peak expired CO_2_, kurtosis, and time to peak CO_2_ after injection of IVSB.

Statistical analysis

For the laboratory study, associations between expired CO_2_ parameters and cardiac output were examined graphically, except for mean expired CO_2_, which was compared using one-way analysis of variance (ANOVA) on ranks (non-parametric) with Steel-Dwass test for pairwise comparisons. Visual inspection from histograms was used to evaluate distributional properties before analysis. For the clinical study, associations between CO_2_ parameters and pump flow were assessed multiple ways. First, Pearson’s correlations (with 95% confidence intervals [95% CI]) were calculated between CO_2_ parameters and pump flow, with time to peak transformed before correlational analysis. For more direct comparison with laboratory study, associations between CO_2_ parameters and pump flow were also assessed using t-tests or non-parametric Mann-Whitney tests, comparing lower and higher flow rates (estimated using median split). A p-value of <0.05 was considered statistically significant. Analyses were conducted in JMP Pro 15 (SAS Institute Inc., Cary, NC). For clinical study, a sample of 18 patients would be able to detect correlations of r = 0.6 that are significantly greater than 0 and would be able to detect group differences in mean expired CO_2_ of 7 mmHg (assuming SD = 5).

## Results

Laboratory study

**Figure 1:**
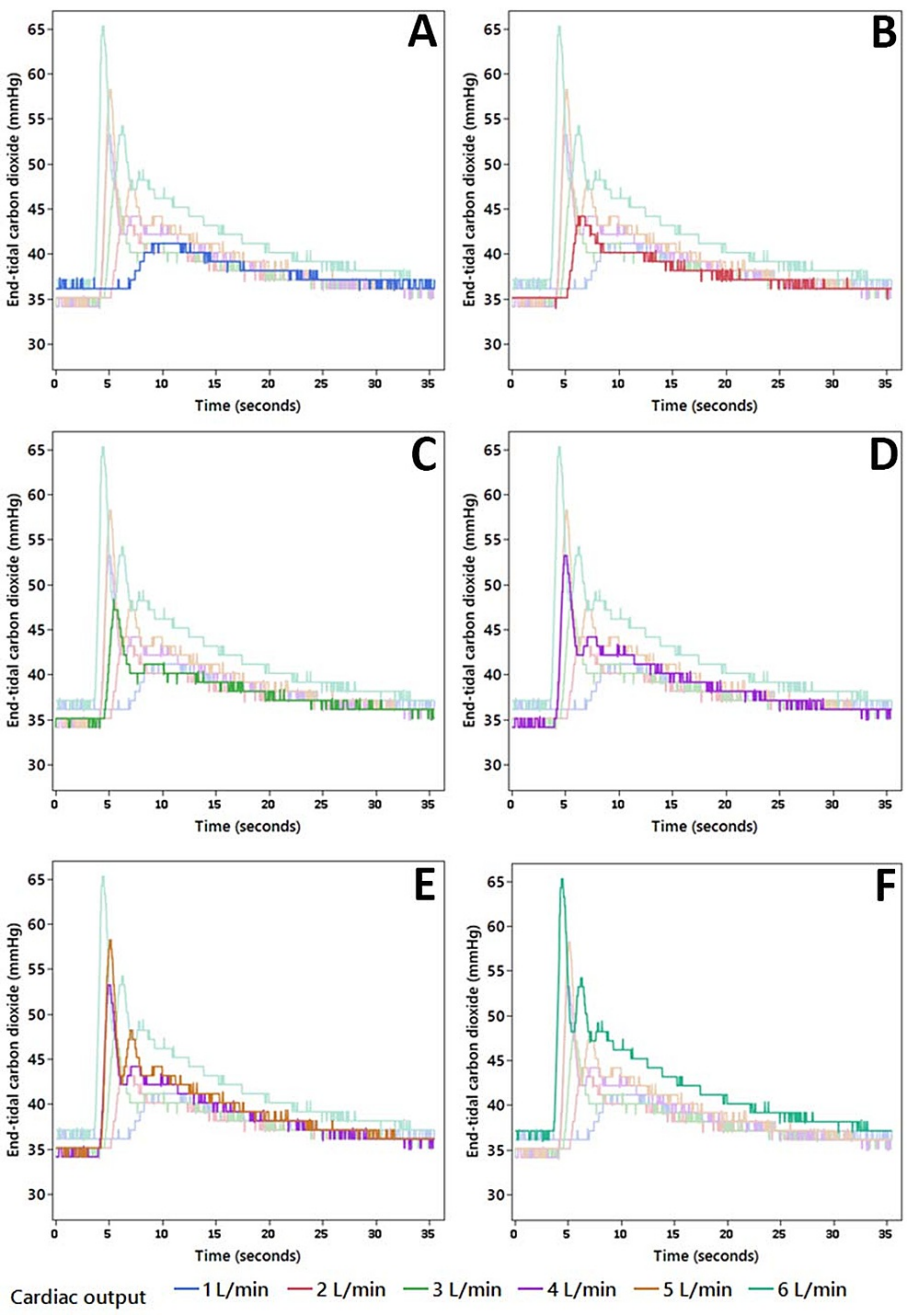
End-tidal carbon dioxide response curve to intravenous sodium bicarbonate injection The waveform has a typical characteristic that can be analyzed and compared. Each panel highlights one of the six cardiac outputs levels evaluated. The differences among cardiac output (1–6 L/min) etCO_2_ response curves are easily visualized. (A) 1L/min; (B) 2L/min; (C) 3L/min; (D) 4L/min; (E) 5L/min; (F) 6L/min. etCO_2_, end-tidal carbon dioxide

**Figure 2:**
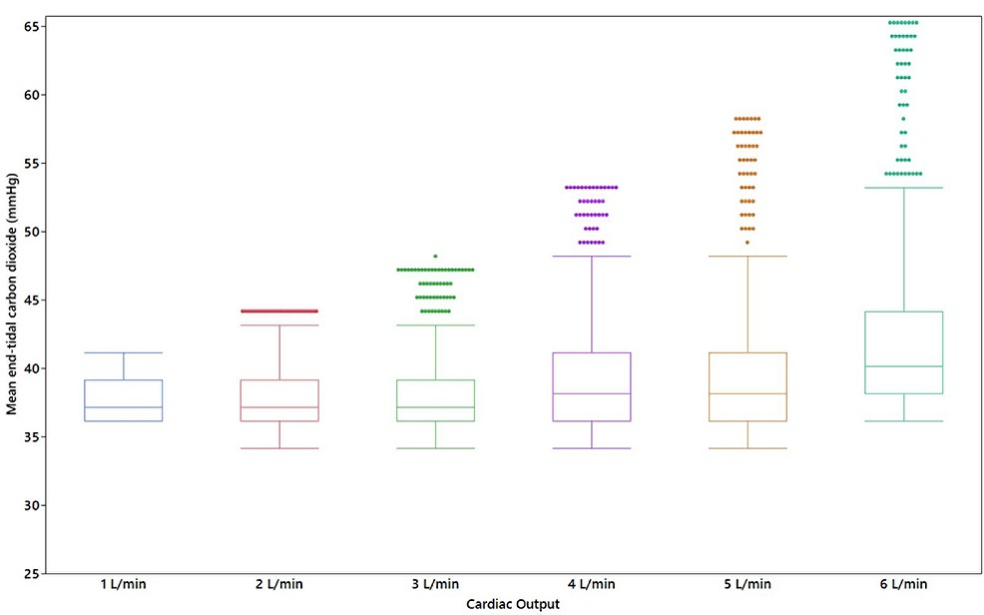
Box plots illustrating the association between mean end-tidal carbon dioxide after intravenous sodium bicarbonate and cardiac output (1–6 L/min) on a cardiopulmonary bypass model High cardiac output (5–6 L/min), moderate cardiac output (3–4 L/min), and low cardiac output (1–2 L/min).

**Figure 3:**
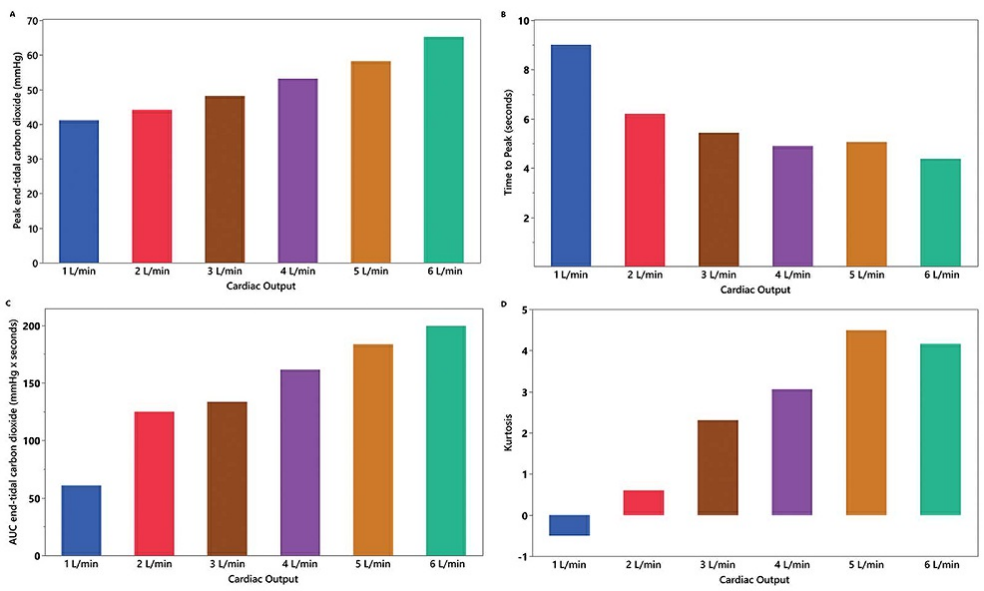
Graphical visualizations The associations between peak end-tidal carbon dioxide (A), time to peak (B), area under the curve, AUC (C), and kurtosis (D) after intravenous sodium bicarbonate and cardiac output (1–6 L/min) on a cardiopulmonary bypass model.

Each administration of IVSB resulted in a transient increase in expired CO_2_ (Figures 1A-1F). The association between mean expired CO_2_ and cardiac output is shown in Figure 2. There was a statistically significant association between mean expired CO_2_ and cardiac output (χ^2^(5) = 1307.9, p < 0.001). Higher mean expired CO_2_ levels were associated with increasing cardiac output. In posthoc pairwise analysis, nearly all cardiac outputs were statistically significantly different from other output levels (p < 0.001); except for comparison between 2 L/min, and 3 L/min (p = 0.21). Other parameters that described the characteristics of the waveform created by IVSB are shown in Figures 3A-3D. Higher peak expired CO_2_, faster time to reach peak, higher AUC, and greater kurtosis of peak were observed at higher cardiac output levels.

Clinical Study

**Figure 4:**
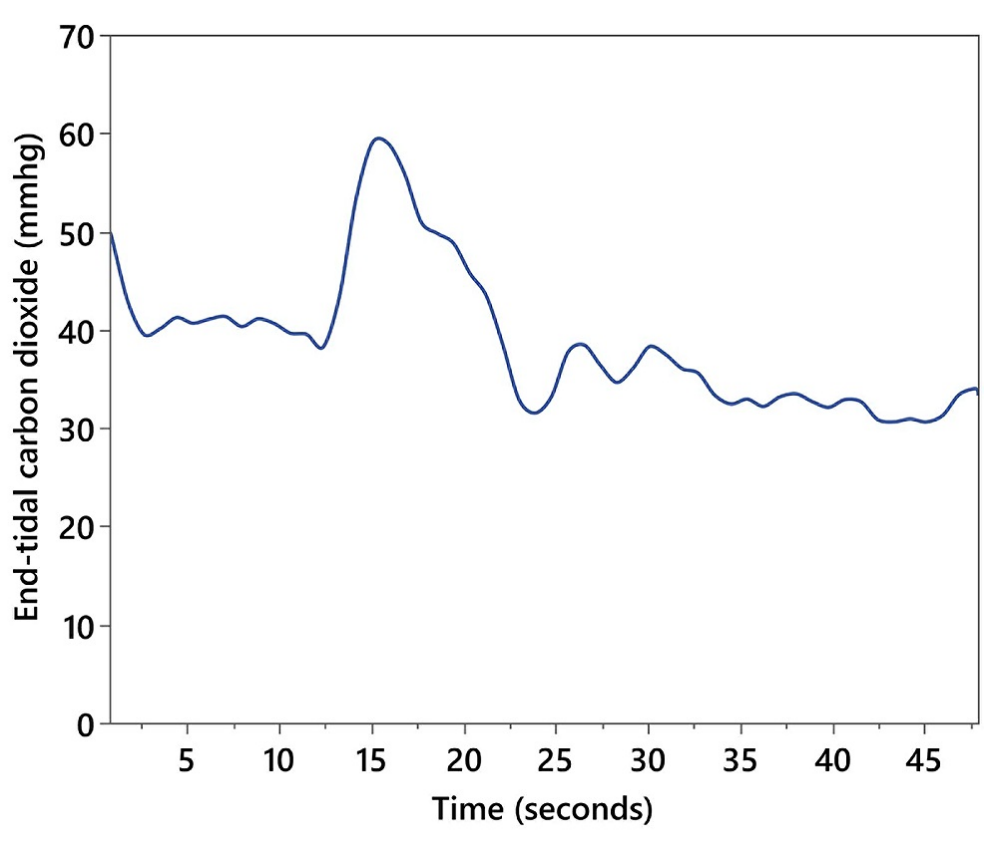
Single patient waveform of end-tidal carbon dioxide response curve to intravenous sodium bicarbonate

**Figure 5:**
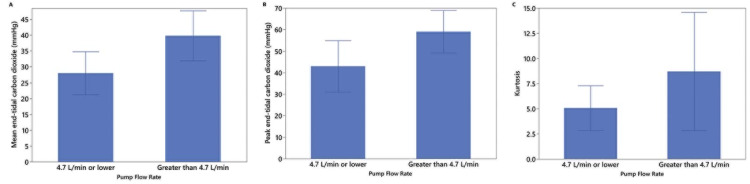
Group differences Group differences in mean end-tidal carbon dioxide (A), peak end-tidal carbon dioxide (B), and kurtosis (C) between cardiopulmonary bypass flow rate at 4.7 L/min or lower and greater than 4.7 L/min (median split). There were statistically significant group differences.

A total of 18 patients were enrolled in the study: 16 patients had a single measurement, one patient had two measurements (when a second dose of IVSB was used for clinical indications), and one patient had one bolus of IVSB while on full bypass and a second measurement during antegrade cerebral perfusion via an axillary cannulation. There were 12 males, and the mean age was 58.6 years (range: 23-77 years). Pump flows ranged from 0.46 to 5.8 L/min. After each injection, a characteristic expired CO_2_ waveform was recorded (Figure 4). Pump flow showed moderate correlations with both mean (r = 0.55, 95% CI: 0.14-0.80) and peak expired CO_2_ (r = 0.51, 95% CI: 0.09-0.78), as well as with kurtosis (r = 0.39, 95% CI: -0.07 to 0.71). Correlation with time to peak was low (r = -0.1, 95% CI: -0.45 to 0.43). Median pump flow was 4.7; this value was used to dichotomize data to compare mean differences between higher and lower cardiac output. Mean (t(18) = 2.5, p = 0.023; Figure 5A) and peak expired CO_2_ (t(18) = 2.4, p = 0.028; Figure 5B) significantly differed between cardiac output levels, with higher mean and peak expired CO_2_ associated with higher cardiac output. The association between kurtosis and CPB flow group (t(18) = 1.9, p = 0.250; Figure 5C) did not reach statistical significance (p = 0.250). There was also no statistically significant association between pump flow rate and time to peak expired CO_2_ (χ^2^(1) = 0.14, p = 0.704).

## Discussion

This study confirmed a correlation between peak CO_2_ release after IVSB administration and bypass flow rate in a bench model of CPB and in humans undergoing surgical procedures while on CPB. Additionally, change in mean CO_2_ and the sharpness of the peak (kurtosis) correlated with the pump flow. In both the bench model and the human trial, the time to peak CO_2_ did not correlate with pump flow. Pump flow while on CPB is cardiac output. The consistency of the correlations between the bench model and clinical trial suggests that the relationship is valid and robust. In the clinical trial, the relationship between flow rate and CO_2_ release was strongest at lower flow rates, equivalent to a low cardiac output state.

Expired or etCO_2_ levels are determined by the balance of tissue production of CO_2_ and ventilation. Increasingly, cardiac output, which parallels pulmonary blood flow, has been recognized as an additional determinant of expired CO_2_, especially acute changes in cardiac output [8]. Okamoto et al. concluded that the time course and the magnitude of changes in end-tidal carbon dioxide tension (PETCO_2_) following intravenous administration of NaHCO_3_ reflect changes in cardiac output and hemoglobin concentration in anesthetized dogs [9]. Others have shown the relationship between cardiac output and etCO_2_ [10]. An increase in etCO_2_ during cardiopulmonary resuscitation is associated with a return of spontaneous circulation (ROSC) and may predict patients in whom ROSC is unlikely to occur [11]. EtCO_2_ has also been shown to correlate with cardiac output after weaning from CPB and predicts adequate post-bypass cardiac output [12]. Changes in etCO_2_ have also been studied as a way to identify changes in cardiac output in anaphylaxis during general anesthesia [13], hemorrhage [14], and septic shock [15], and as a useful bedside test to predict fluid responsiveness in cardiogenic shock [16].

The indicator-dilution technique is a common way to measure cardiac output and is based on Stewart’s adaption of the original principle described by Fick in 1870 [17]. This method involves the administration of a known quantity of something, such as a cold fluid, heat pulse, colored dye, or other substance, and measuring the appearance of that indicator at a distant site. This is the underlying principle used with pulmonary artery catheters for intermittent or continuous cardiac output measurement. Theoretically, any substance that can be given in a known quantity and measured quickly at a distal site could be used to measure cardiac output. IVSB is rapidly converted to CO_2_. Intubated and ventilated patients in the operating room and intensive care unit have continuous exhaled CO_2_ measured as part of standard care. If changes in exhaled CO_2_ after administration of IVSB are correlated with cardiac output, then cardiac output and subsequent changes over time could be measured without further invasive monitoring.

The clinical measurement of cardiac output currently requires an invasive monitor such as a pulmonary artery catheter or an arterial line, whereas intubated patients have routine continuous CO_2_ monitoring. IVSB is sometimes required during major surgery, including cardiac surgery, on bypass, and during the postoperative period. Administration of intermittent boluses of IVSB is usually benign, and it may be indicated to help correct metabolic acidosis. If data from the change in exhaled CO_2_ after IVSB administration can be correlated with cardiac output, this would be an additional option to evaluate cardiac output.

This was a proof-of-concept study and as such has limitations. CPB was a surrogate for cardiac output, and the technique still needs to be assessed in patients not on bypass. The effects of IVSB on the heart of critically ill patients or in patients with pre-existing acidosis need further elucidation as current evidence is lacking, although research suggests that there is no effect on hemodynamic variables [18,19]. Additionally, there is evidence that hemoglobin levels may affect the rate of bicarbonate metabolism, which may act as a confounder in patients with large variations in hemoglobin concentrations. Additionally, the lack of study of carbonic anhydrase function is a potential limitation. Although we believe its effect should be minimal compared to other factors, different disease processes and interventions may affect enzyme concentration and possible conversion rates of bicarbonate. The concentration and volume of sodium bicarbonate administered were arbitrarily chosen, and dose-finding studies that retain accuracy while minimizing the sodium bicarbonate load may be warranted.

## Conclusions

In this proof-of-concept study, we demonstrated an association between changes in exhaled CO_2_ and CPB flow after the administration of sodium bicarbonate. This technique may have utility in intermittent assessment of cardiac output or improve accuracy when used in conjunction with other continuous output monitors. This novel, minimally invasive indicator-dilution technique warrants further study in a broader range of patients.
